# Activated lymphocytes as a metabolic model for carcinogenesis

**DOI:** 10.1186/2049-3002-1-5

**Published:** 2013-01-23

**Authors:** Andrew N Macintyre, Jeffrey C Rathmell

**Affiliations:** 1 Department of Pharmacology and Cancer Biology, Department of Immunology, Sarah W. Stedman Nutrition and Metabolism Center, Duke University, Durham, NC, 27710, USA

**Keywords:** Cancer, Lymphocyte, Metabolism, Aerobic glycolysis

## Abstract

Metabolic reprogramming is a key event in tumorigenesis to support cell growth, and cancer cells frequently become both highly glycolytic and glutamine dependent. Similarly, T lymphocytes (T cells) modify their metabolism after activation by foreign antigens to shift from an energetically efficient oxidative metabolism to a highly glycolytic and glutamine-dependent metabolic program. This metabolic transition enables T cell growth, proliferation, and differentiation. In both activated T cells and cancer cells metabolic reprogramming is achieved by similar mechanisms and offers similar survival and cell growth advantages. Activated T cells thus present a useful model with which to study the development of tumor metabolism. Here, we review the metabolic similarities and distinctions between activated T cells and cancer cells, and discuss both the common signaling pathways and master metabolic regulators that lead to metabolic rewiring. Ultimately, understanding how and why T cells adopt a cancer cell-like metabolic profile may identify new therapeutic strategies to selectively target tumor metabolism or inflammatory immune responses.

## Review

The mid-twentieth century has been described as the ‘golden age of intermediary metabolism’ [1], with the work of Krebs, Lippman, Crane and others greatly advancing our understanding of cellular metabolic pathways. In the past decade interest in cell metabolism has been rejuvenated in several fields, especially cancer biology and lymphocyte immunology. In cancer biology, this renaissance has been driven by evidence that cancer metabolism presents an underexploited therapeutic target. Immunologists have been drawn to metabolic studies with the realization that the metabolism of T lymphocytes (T cells) is intimately tied to immunity [2]. Functionally, T cells and tumors have little in common; the former protects against invasive pathogens, the latter is a diseased tissue characterized by the accumulation of abnormal cells. However, both T cells and cancer cells have strong proliferative signals and undergo metabolic reprogramming during their respective life cycles, and there exist clear functional and mechanistic similarities between the reprogramming events in each cell type. These similarities make lymphocyte metabolic reprogramming a useful model with which to discover how and why tumors rewire their metabolism. The purpose of this review is to highlight and discuss the similarities and distinctions in how T cells and tumor cells solve similar metabolic problems.

### T lymphocyte activation: a key lifestyle switch

Because of its inherently destructive nature, the immune system must be maintained in a quiescent state. To provide protection from pathogens, however, it must remain capable of rapid responses and effector function. This challenge is solved with a diverse pool of naïve lymphocytes that can quickly activate to produce a large, clonal pool of rapidly proliferating effector T cells. Naïve T cells express near-unique T cell antigen receptors (TCR) that are randomly generated through V(D)J recombination and pre-selected to recognize foreign antigens presented on major histocompatibility complexes (MHC). These naïve cells continually circulate the blood and lymphatic system sampling MHC-peptide complexes. Upon encounter with an antigen-presenting cell (APC) and cognate antigen, the T cell ceases to migrate, forming a prolonged contact with the APC. This induces sustained signaling through the TCR and other co-receptors, inducing T cell activation, proliferation and differentiation into effector cells. These effectors rapidly accumulate and migrate to sites of inflammation, ultimately clearing the invader [3].

Activation therefore simultaneously places T cells under several types of stress: they must proliferate rapidly; they must synthesize large amounts of effector proteins; and they have to prepare to enter a heterogeneous and potentially hypoxic, nutrient poor environment. Each of these stressors has a significant metabolic aspect reminiscent of the classic cancer metabolism paradigm: proliferation and anabolism require energy, biosynthetic building blocks and reducing equivalents, while nutrient stress and hypoxia both potentially limit metabolic flux by restricting metabolite access and oxygen. With similar metabolic demands and stresses, it is not surprising that these diverse cell types respond by adopting a similar metabolic profile.

### A common metabolic solution: aerobic glycolysis

**Figure 1:**
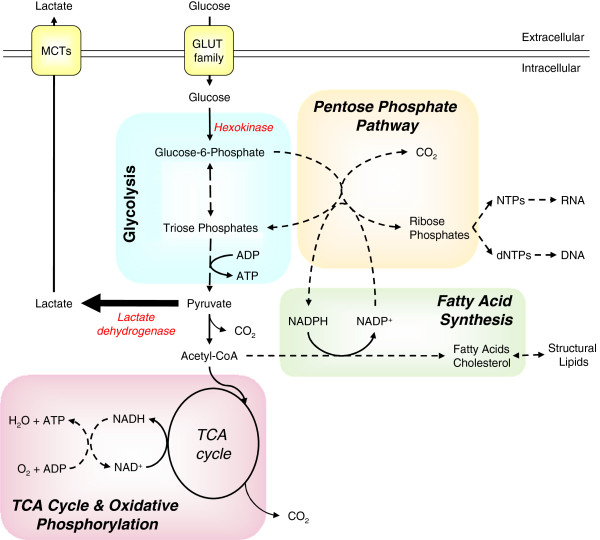
**Major metabolic fates of glucose in highly proliferative cells. **Glucose is taken into the cell by GLUT family transporters and then phosphorylated by hexokinases, trapping it within the cell as glucose-6-phosphate (G6P). G6P can be catabolized via glycolysis or used as a carbon donor for the synthesis of riboses via the pentose phosphate pathway (PPP). Catabolized G6P generates pyruvate plus small quantities of ATP, with much of the resultant pyruvate being converted to lactate by lactate dehydrogenase and then secreted through mono-carboxylic transporters (MCT). The remaining pyruvate is converted to acetyl-CoenzymeA (acetyl-CoA) by pyruvate dehydrogenase and used either as fuel for ATP production via the tri-carboxylic acid (TCA) cycle and oxidative phosphorylation or converted to fatty acids to generate structural lipids. At various points during glycolysis and the TCA cycle reaction intermediates can be removed to provide carbon for amino acid biosynthesis (not shown).

Three metabolic pathways are central to ATP production in proliferative lymphocytes and cancer cells: glycolysis, the tri-carboxylic acid (TCA) cycle and oxidative phosphorylation (OXPHOS). While the TCA cycle does not directly generate ATP, it is inexorably linked to OXPHOS, providing several metabolic inputs to drive ATP production. In addition, intermediate metabolites from both the TCA cycle and glycolysis can be used as carbon sources for catabolic pathways producing cholesterol, lipids, ribose, and other biosynthetic molecules (Figure 1) [4]. Resting or non-proliferative cells often rely on mitochondrial lipid β-oxidation. Proliferative cells, in contrast, generally decrease lipid oxidation and instead conserve lipids to support cell growth [5]. 

For mammalian cells that lack significant intracellular nutrient stores, extracellular glucose uptake represents a major carbon and energy source. Glucose is transported through facilitative glucose transporters and phosphorylated by hexokinases to initiate metabolic pathways and prevent its exit. Glucose-6-phosphate (G6P) is primarily metabolized through glycolysis or the pentose phosphate pathway (PPP). Glycolysis provides a small net ATP gain per glucose molecule consumed and yields pyruvate which can then either be: i) reduced to lactate by lactate dehydrogenase (LDH), concomitantly restoring NADH to NAD+, ii) converted to alanine by alanine aminotransferase, simultaneously converting glutamine to α-ketoglutarate, or iii) converted to acetyl-CoenzymeA (acetyl-CoA) in the mitochondria to be oxidized via the TCA cycle, generating large amounts of ATP via OXPHOS (respiration). Most non-proliferating cells utilize this latter pathway when oxygen is available in a process termed the Pasteur effect.

Not all cells, however, exhibit the Pasteur effect and cease lactate production under aerobic conditions. In the early 20th century, Otto Warburg observed that many tumor cells and tumor sections continued lactate secretion in the presence of oxygen [6]. This metabolic program is referred to as aerobic glycolysis, differentiating it from the obligatory fermentation of glucose to lactate that occurs under anaerobic conditions where no oxygen is available to fuel OXPHOS. Warburg postulated that the switch towards aerobic glycolysis arose from faults in respiration and that such defects were the primary cause of cancer [6,7]. While his observations stand, his proposed mechanism for aerobic glycolysis has now largely been discounted following studies demonstrating that cancer cells often have grossly normal respiratory function [8-10] and, indeed, can exhibit elevated rates of respiration [11]. Nevertheless, mitochondrial mutations are associated with some cancers and the relationships between aerobic glycolysis, mitochondrial function and tumorigenesis remain controversial [12].

**Figure 2:**
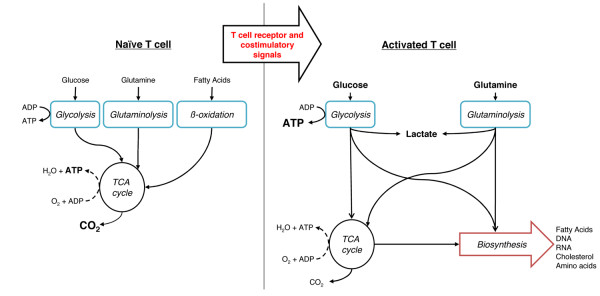
**T cell activation results in metabolic reprogramming. **Naïve T cells have an oxidative metabolism, using glucose, glutamine, and fatty acids as fuel sources. The majority of ATP is generated via oxidative phosphorylation. Following activation by stimulation of the T cell receptor and co-receptors, the cells adopt a metabolic profile that resembles the metabolism of many cancer cells, consuming large quantities of both glucose and glutamine but performing relatively little oxidative phosphorylation. The majority of glucose-derived carbon is secreted as lactate, with the remainder being used for biosynthesis.

Similar to his observations of aerobic glycolysis in cancer cells, in 1958 Warburg also found that stimulated leukocytes become highly glycolytic [13]. Subsequent reports in the 1970s to 1990s, using lectin-stimulated rat thymocytes and lymphocytes, also showed lymphocytes become glycolytic upon activation. Together, these studies demonstrated that resting lymphocytes obtain most of their ATP by OXPHOS of glucose, amino acids, and lipids. However, within hours of stimulation, lymphocytes begin to increase glucose uptake up to forty- or fifty-fold and to secrete most of the glucose-liberated carbon as lactate [14] (Figure 2). In parallel, lymphocytes increase oxygen consumption by around 60% [15-19]. These data have subsequently been confirmed using purified T cell populations stimulated with antibodies that trigger the TCR complex and associated co-receptors [20,21]. Importantly, this increase in aerobic glycolysis precedes and has been shown to be essential for the growth and proliferation of stimulated T cells [21-23]. 

Cancer cells and T cells are not metabolically unique, and the induction of aerobic glycolysis has also been reported during proliferation of other non-transformed cells. For example, a similar phenotype is also observed in both intestinal cells and fibroblasts during logarithmic growth [4,24]. However, few other cell types have shown such a distinct and acute induction of aerobic glycolysis from a near proliferative and metabolic standstill. T cell activation, therefore, provides a unique model to explore how and why metabolic rewiring occurs in cancer cells.

#### Aerobic glycolysis supports rapid proliferation

The metabolic needs of T cells change dramatically upon activation. Before encountering pathogens, resting T cells require only sufficient energy to support basal cellular needs and replacement biosynthesis. After activation, T cells undergo a transient period with little cell growth and then begin to rapidlyÂ grow and divide. T cells specific for a given MHC-antigen complex are rare [25,26], so clonal expansion must rapidly expand these small populations of hundreds of cells to the tens or hundreds of millions of cells necessary for protection. Remarkably, activated T cell doubling times of 4 to 6h have been observed *in vitro*[27], with even faster doubling rates reported *in vivo*[28,29]. Activated T cells, therefore, have a tremendous need for both ATP [30] and biosynthetic capacity to synthesize new proteins, lipids, and nucleic acids.

While a hallmark of cancer is cell cycle deregulation, there is little propensity for tumor cells to adopt increasingly rapid rates of cell division like activated T cells. Indeed, the majority of cells within a solid tumor may be in a state of G1 cell cycle arrest [31]. Extensive clinical studies have shown that although cell cycle length in tumors is more diverse than non-cancerous tissue, the median S-phase length across all tumor types is around 10 h [32] and, counter-intuitively, there is no clear relationship between proliferative ability and tumor aggressiveness [33]. Still, proliferation of cancer cells must exceed cell death to allow tumor growth. Thus, with the exception of an alternate glycolytic pathway in which tumor cells may bypass pyruvate kinase to convert phosphoenol pyruvate to pyruvate, and yield no net gain of ATP [34], activated T cells and tumor cells harness aerobic glycolysis to provide ATP and biosynthesis for proliferation.

#### Advantages of aerobic glycolysis: rapid ATP production

In contrast to OXPHOS, glycolysis is energetically inefficient, theoretically yielding only two molecules of ATP per glucose molecule consumed compared to up to thirty-six if fully oxidized. This is not a trivial issue as cancer cells have been shown to possess additional, unused respiratory capacity [8,35,36]. Thus, cancer cells do not increase glycolysis solely because their capacity for OXPHOS is saturated. Rather, aerobic glycolysis and basal OXPHOS provide sufficient energy to support the cell survival and growth demands of cancer cells and activated T cells.

One energetic advantage of adopting aerobic glycolysis as a primary metabolic program is the speed at which ATP can be regenerated. While OXPHOS yields more ATP than glycolysis, there is a trade-off between yield and speed [37,38]. Indeed, as described by Koppenol and Bounds [39], Warburg and colleagues observed this phenomena as early as 1923, reporting that for every one molecule of glucose oxidized by respiration, twelve are metabolized by glycolysis. Increased glycolysis can boost ATP production rate by two-thirds, provided cells are not concerned with efficiency. While wasteful, therefore, the speed of aerobic glycolysis offers a selective advantage both to tumor cells competing against other cells within the same environment [37,40], and to T cells racing to suppress invading pathogens.

#### Advantages of aerobic glycolysis: biosynthesis

Beyond ATP production, glycolysis and the TCA cycle form the nexus for many biosynthetic processes. Carbon intermediates derived from glycolysis and the TCA cycle are used for the generation of amino acids, lipids, cholesterol and nucleotides. A major function of aerobic glycolysis, therefore, is to provide sufficient intermediates to fuel biosynthesis for proliferation and growth. Indeed, increased glucose uptake can enhance T cell responses and growth *in vivo* as mice transgenically overexpressing the glucose transporter GLUT1 in T cells accumulate effector T cells with age [22,41] and GLUT1 overexpression is correlated with poor prognosis in a variety of cancers [42].

Rapid glucose uptake fuels both glycolysis and the PPP, each of which provides numerous metabolites to support cell growth. Glycolysis is a major source of serine synthesis as well as pyruvate that can either be converted to lactate to replenish NAD+ or can be transported into the mitochondria to enter the TCA cycle as acetyl-CoA. From the TCA cycle, citrate can exit to the cytosol to provide a basis for lipid synthesis [21,43]. Under hypoxic conditions, glutamine can undergo reductive carboxylation to provide a reverse flow through the TCA cycle as a source of lipogenesis in both cancer cells and in CD8+ T cells [44]. Notably, both tumor cells [45] and lectin-stimulated lymphocytes [46,47] perform extensive *de novo* synthesis of lipids, and only limited lipid β-oxidation. In addition to *de novo* lipogenesis, aggressive cancer cell lines and primary tumors also perform extensive lipid remodeling, in part due to elevated monoacylglycerol lipase activity [48]. Tumor lipid metabolism can be further enhanced by Akt-driven expression of the low-density lipoprotein receptor (LDLR), which increases cholesterol intake and promotes cell growth [49]. The relative importance of each of these pathways to lymphocyte lipid metabolism has yet to be determined.

The PPP provides nicotinamide adenine dinucleotide phosphate (NADPH) reducing potential and generates ribose sugars that can be directed into TCA cycle intermediates and into purine, pyrimidine and aromatic amino acid synthesis pathways. The PPP are strongly induced in T cell activation [21] and can be important in cancer; indeed U-C14 glucose tracer experiments have suggested that in some tumor types over 80% of the nucleotides in DNA and RNA are synthesized from glucose-derived carbon [50,51]. Upregulation of the PPP is facilitated, in part, by increased enzyme expression. Activated T cells increase expression of PPP enzymes and high levels of PPP enzyme activity have been reported in metastatic tumor cells [52]. For example, glioblastoma expression of the transketolase TKTL1, the key enzyme linking the PPP to glycolysis, directly correlates with tumor severity in the clinic [53].

NADPH is a critical reducing agent in the synthesis of fatty acids and cholesterol as well as maintaining cellular redox status and control reactive oxygen species (ROS) produced by OXPHOS [54]. While some degree of ROS is beneficial for both T cell activation [55] and tumor development [56], excessive ROS leads to oxidative organelle damage and the induction of apoptosis. Strategies that drive cancer cells to increase the OXPHOS-glycolysis ratio, for example by increasing pyruvate dehydrogenase activity to drive mitochondrial conversion of pyruvate to acetyl-CoA, decrease both proliferation and growth [57]. Similarly, glucose restriction of activated lymphocytes induces an increase in OXPHOS, a drop in glycolysis, and an inhibition of proliferation [20,58]. In proliferating cells efficient OXPHOS should, therefore, be balanced by high PPP flux to prevent overloading the demand for NADPH.

#### Advantages of aerobic glycolysis: adaptation to the environment

Glycolysis and the TCA cycle are amphibolic and supply both ATP and intermediates to multiple pathways to potentially support cells under stress conditions. Indeed, we have shown that high rates of glycolysis can be protective against apoptosis [59,60]. A high rate of metabolic flux makes it thermodynamically less costly to redirect intermediates down different pathways, that is, high metabolic flux permits rapid rerouting of metabolites [61-63]. This control sensitivity may permit a faster response to specific nutrient deprivation as cells enter potentially nutrient-poor environments. This may explain why the rate of glucose consumption in both activated T cells and many tumor types appears in excess of that required to meet either the biosynthetic or energetic demands of the cell [64].

Further, glycolysis is not oxygen dependent, and so adopting a glycolytic metabolism can prepare cells for entry or survival in a hypoxic environment. Even after vascularization, solid tumors feature extensive hypoxic domains [65]. Similarly, lymph nodes [66], spleen [67], tumors, dermal/surgical wounds [68] and other regions frequented by activated lymphocytes contain extensive areas of low oxygen tension. Adaption of a highly glycolytic metabolism with low oxygen dependency may help both tumors and lymphocytes survive and proliferate during low oxygen availability.

### Common mechanisms drive glycolytic reprogramming in T cells and tumors

#### Transporter expression and izozyme switching

A limiting step in glucose metabolism is the rate at which glucose can be captured and trapped within the cell. There are two major glucose transporter families, the Na+/glucose linked transporter (SGLT) symporters, and the GLUT family of passive transporters. Fourteen mammalian GLUT family transporters have been identified [69] and the major glucose transporters in lymphocytes appear to be GLUT1 and GLUT3, the expression levels of which increase significantly following activation [70]. Facilitated diffusion of glucose by the GLUTs requires a glucose gradient across the extracellular membrane. This so-called glucose sink is maintained by hexokinase phosphorylation of intracellular glucose. Following T cell activation, hexokinase activity increases significantly [71] and T cells undergo a switch in HK isozyme expression from HKI to HKII [72,73]. While both HKI and HKII both feature two potential catalytic domains, in HKI one of these is non-functional, thus HKII has a higher Km for both glucose and ATP compared to HKI [74]. Second, signals from the TCR and co-receptors drive HKI and HKII to bind mitochondria at porin (ATP-exporting) complexes [75]. This close coupling of HK and mitochondria provides HKII with access to a large pool of ATP.

**Figure 3:**
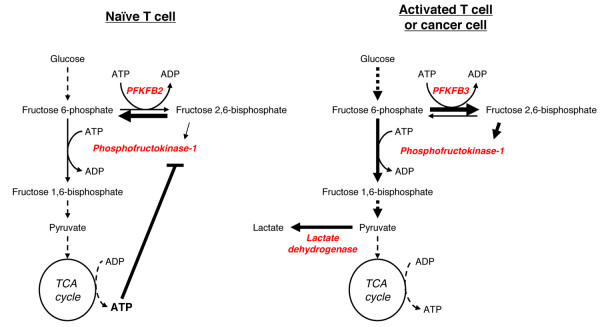
**Glycolytic isozyme switching promotes high rates of glycolysis. **Activated T cells, cancer cells and other highly proliferative cells express different glycolytic isozymes in comparison to quiescent cells, increasing glycolytic flux. One key step in glycolysis is the phosphorylation of fructose 6-phosphate by phosphofructokinase-1 (PFK-1). PFK-1 is allosterically activated by fructose 2,6-bisphosphate and allosterically inhibited by ATP. Both activated T cells and tumor cells express isoform 3 of the bifunctional enzyme 6-phosphofructo-2-kinase/fructose-2,6-bisphosphatase (PFKFB). In contrast, naïve T cells express PFKFB isoform 2. PFKFB3 differs from PFKFB2 in that it has low phosphatase activity, leading to the accumulation of fructose 2,6-bisphosphate and localized depletion of ATP. This results in increased PFK-1 activity and higher rates of glycolysis.

Following lectin stimulation, lymphocytes also switch expression of other glycolytic isozymes. This includes induction of pyruvate kinase M2 (PKM2), LDH-A4, and enolase I [21,73]. These changes in expression are associated with increases in maximal glycolytic enzyme activity [16,72], and the relieving of allosteric inhibition that would otherwise limit glycolytic flux. One example of this is the regulation of the glycolytic enzyme 6-phosphofructo-1-kinase (PFK1), a key regulatory enzyme in glycolysis (Figure 3). PFK1 is allosterically inhibited by ATP and allosterically activated by fructose-2,6-bisphosphate (F26P2). F26P2 is generated by the bifunctional enzyme 6-phosphofructo-2-kinase/fructose-2,6-bisphosphatase (PFKFB), and in naïve lymphocytes PFKFB isoform 2 predominates. However, following activation T cells express large quantities of PFKFB isoform 3 [76,77]. PFKFB3 has a very low phosphatase activity compared to PFKFB2 [78], and so this isozyme switch enhances PFK1 flux by both increasing F26P2 and depleting ATP. 

Cancer cells also show a general increase in glycolytic enzyme activity and expression of specific isozymes. This includes expression of HKII, LDH-A and PFKFB3 [52,79,80]. Tumor cells express PKM2, but there is now strong evidence that this is largely in the metabolically inactive, dimeric form, rather than the active tetramer [81]. In many tumor cells PKM2 activity is further inhibited by direct tyrosine phosphorylation and by the binding of phosphotyrosine containing peptides, both of which restrict cofactor binding. Reduced PKM2 activity enhances aerobic glycolysis and tumor growth [82,83]. Cascades of tyrosine phosphorylation are central to T cell activation; however, it has yet to be determined if these cascades result in PKM2 inhibition. Recent reports in tumor cells have demonstrated that PKM2 can be selectively degraded in an acetylation-dependent fashion at times of high glucose availability [84], allowing additional glycolytic intermediates to be used for biosynthesis. Phosphoenol-pyruvate flux through a non-ATP generating pathway may then sustain rapid pyruvate generation while preventing ATP-driven feedback inhibition of glycolysis [34]. This regulatory loop for PKM2 may represent a further mechanism to maintain high rates of glycolytic flux, but this has yet to be examined in activated lymphocytes.

### Beyond glucose metabolism: glutamine

Glutamine has multiple metabolic fates, being used for ATP regeneration, anaplerosis of the TCA cycle, and redox regulation. Within the cell glutamine is readily converted to glutamate by glutaminase. Glutamate is used together with cysteine and glycine to generate glutathione, is used for lipid synthesis through reductive carboxylation under hypoxia, and is a major nitrogen donor during purine and pyrimidine synthesis. Naïve lymphocytes utilize glutamine as a primary oxidative fuel for ATP generation. Following T cell activation, cMyc greatly increases the expression of glutaminolysis enzymes and the rate of glutamine uptake [15,21]. After conversion to glutamate, glutamate dehydrogenase generates α-ketoglutarate to support the TCA cycle. Notably, while the early stages of lymphocyte activation do not require glutamine, subsequent proliferation and the expression of effector cytokines following TCR stimulation correlate directly with glutamine availability [85-87], and there is clinical evidence to suggest that glutamine availability can be a limiting factor in lymphocyte activation during inflammatory responses [88-90].

Many tumor types exhibit high rates of glutamine consumption relative to non-transformed cells [91-93]. Cancers driven by oncogenic cMyc, for example, become highly dependent on glutamine [94,95] and can be exquisitely sensitive to glutamine deprivation [96]. Other tumors, however, can exhibit little sensitivity to glutamine deprivation [93,97-99]. This resistance to glutamine deprivation may relate to the induction of glutamine synthase in these cells, and so although less dependent on exogenous glutamine, they still exhibit high rates of glutamate flux. Also, expression of pyruvate carboxylase can allow glucose-derived pyruvate to convert to oxaloacetate to support the TCA cycle and maintain export of citrate for lipid synthesis through anapleurosis [100]. Given these potential differences, activated T cells may represent a better metabolic model for primarily glutamine-dependent tumors.

### Common signaling events drive metabolic reprogramming

The cancer metabolism phenotype is ultimately initiated by oncogenic signaling events that induce metabolic gene expression and stimulate aerobic glycolysis. Importantly, T cell receptor and co-receptor engagement are now well understood and activate many of these same signaling pathways (see Smith-Garvin *et al.*, 2009, for a detailed review [101]). Briefly, the TCR is associated with several CD3 accessory chains and when the TCR is engaged, tyrosine phosphorylation of accessory chains recruits kinases and scaffold proteins. This recruitment, along with co-stimulation, triggers localized stimulation of three signaling pathways: calcium flux, MAPK (ERK/p38) signaling, and phosphatidylinositol-3,4,5-trisphosphate (PI(3,4,5)P3) signaling. Autocrine and paracrine cytokine signaling loops induce further PI(3,4,5)P3 and MAPK activation, along with JAK/STAT signaling. Notably, several of the downstream targets of these pathways regulate key metabolic regulators, with mutations in components downstream of these pathways strongly implicated in oncogenesis. Identifying the specific signaling pathways in activated T cells that induce metabolic reprogramming is therefore informative in identifying the oncogenes involved in driving the same processes in tumors.

#### PI3K, PTEN, Akt and mTORC1

PI(3,4,5)P3 is generated by phosphatidylinositol-3-kinase (PI3K) and depleted by phosphatases such as the tumor suppressor, PTEN (phosphatase and tensin homologue deleted on chromosome 10). Both sides of this signaling equilibrium can impact cancer, as activating PI3K and disrupting PTEN mutations frequently promote constitutive signaling through PI(3,4,5)P3-dependent pathways [102]. Of the downstream targets for PI(3,4,5)P3 signaling, the best described is Akt, an established metabolic regulator in both tumors and lymphocytes. In hematopoietic cells and naïve T cells, the expression of a constitutively active Akt leads to increased GLUT1 surface localization, improved coupling of HKII to the mitochondria and increased rates of glycolysis [20,103,104]. Similarly, in tumor models Akt drives cells towards aerobic glycolysis and makes cells highly dependent on exogenous glucose for survival [105].

Akt promotes aerobic glycolysis by direct phosphorylation and activation of glycolytic enzymes, such as PFK2 [106], by phosphorylation of TBC1D1/4 to regulate GLUT1 trafficking, and by regulating several transcription factors (reviewed in detail by Manning and Cantley, 2007) [107]. Further, Akt is able to activate mTORC1 (mammalian target of rapamycin complex 1) via phosphorylation of upstream regulators PRAS40 and TSC2. mTORC1 is a key driver of anabolic metabolism. Indeed, activating the PI3K/Akt pathway can be considered a key regulator of glucose metabolism in both T cells and cancer [108]. Inhibition of this pathway in T cells is potently immunosuppressive and leads to generation of tolerant or regulatory T cells rather than effectors. Given the frequency of cancer-associated mutations in this pathway, delineating how PI(3,4,5)P3 signaling leads to metabolic reprogramming in lymphocytes may provide a unique opportunity to understand the regulation of cancer metabolism.

#### MAPK and HIF1α

The multifactorial roles of the mitogenic ras-MAPK signaling pathways in cancer have been extensively reviewed recently [109-111]. MAPK have multiple roles in metabolic regulation in both tumors [112] and during T cell activation [71,87]. One mechanistic role of recent interest is MAPK regulation of hypoxia inducible factor 1α (HIF1α). HIF1α is a heterodimeric transcription factor that induces gene expression in response to hypoxia. HIF1α induces the expression of many glycolytic genes, and HIF1α can be a key mediator of the Pasteur effect in normal cells [113]. HIF1α protein levels are elevated without the need for hypoxia by PI(3,4,5)P3 signaling through mTOR and other pathways. Activated T cells and many tumor cells, therefore, can exhibit elevated levels of HIF1α. MAPK, however, also play a key role in enhancing HIF1α transcriptional ability, by enhancing HIF1α interactions with transcriptional co-factors [114].

HIF1α is not strongly expressed in normal tissues under normoxic conditions and presents a potential therapeutic target to selectively suppress tumor glucose metabolism. In support of this strategy, several studies have reported that HIF1α null tumor xenografts show reduced growth, while overexpression of xenograft HIFα promotes increased growth [115]. Curiously, and in contrast to these data, HIF1α−/− T cells exhibit normal proliferative and initial metabolic responses to TCR and co-receptor stimulation [116,117]. Instead, the impact of HIF1α loss is only apparent when activated HIF1α−/− T cells are subsequently skewed to different cell fates. HIF1α−/− CD4+ T cells are unable to form interleuken-17 (IL-17) producing T helper cells, which are highly glycolytic. Instead, HIF1α−/− T cells become immunosuppressive regulatory T cells in which lipid metabolism, not glycolysis, is the major metabolic program [41,117]. The role of HIF1α in metabolic regulation is therefore limited during T cell activation. Determining the signaling context by which T cell skewing directs HIF1α regulation of metabolism may, however, be informative in determining how HIF1α functions in tumors.

#### JAK/STATs and the PIM kinases

T cell activation induced metabolism is maintained by sustained signaling from IL-2 and other cytokines acting on common gamma chain (γc) cytokine receptor complexes. This effect is in part mediated by direct and STAT5 driven PI(3,4,5)P3/Akt signaling [118,119]. However, additional STAT driven, Akt-independent, signaling events also play a role. Of note, JAK/STAT3 signaling in lymphocytes induces the expression of the PIM family of kinases, which themselves can promote glycolytic metabolism [120].

PIM kinases are constitutively active [121] and are potent oncogenes, being induced by, and synergizing with, the transcription factor cMyc in several cancer types [122]. In addition, persistent STAT3 signaling is common in many tumor types. While oncogenic STAT3 mutations have not been reported, aberrant STAT3 signaling can arise from inactivation of the STAT-suppressing suppressor of cytokine signaling (SOCS) proteins or by elevated activation of JAKs [123]. The γc-receptor-directed maintenance of activated T cell metabolism, therefore, potentially presents a useful tool with which to study the role of STAT-driven, PIM-mediated, regulation of metabolism. Unfortunately, the PIMs share substrate specificity with Akt [120], and are inhibited by the classical PI3K inhibitor LY294002, a compound historically used to study Akt function [124]. The specific role of PIM kinases in metabolic reprogramming is hence unclear. Studies of activated, PIM-null T cells [125] may help resolve this issue.

#### Calcium signaling and AMPK

Immediately after TCR activation there is a coordinated flux of calcium from intracellular stores and also an increase in mitochondrial calcium uptake [126]. These changes stimulate the calcium-activated mitochondrial dehydrogenases that drive the TCA cycle [127]. In addition, calcium flux downstream of the TCR causes a short term phosphorylation of AMP activated protein kinase (AMPK) [128], a master metabolic regulator that promotes catabolic pathways when the ATP-AMP ratio falls. AMPK is activated by binding of AMP and when phosphorylated by CaMKKβ or the tumor suppressor LKB1 [129]. While the metabolic impact of AMPK activation by the TCR has yet to be fully defined, calcium-induced AMPK activity during T cell activation may help to promote an initial phase of oxidative and ATP- generating metabolism. This could prepare T cells to enter a rapid growth phase and to resist the stress of nutrient-deficient conditions. The latter role may be particularly important as AMPK-null T cells show only a limited metabolic phenotype under nutrient-rich conditions, but fail to respond to metabolic stress *in vitro*[130]*. In vivo,* nutrients are potentially limiting in lymph nodes or inflamed tissues, and TCR-induced activation of AMPK may be important to maintain ATP levels and maximize survival, so that T cells can proceed to a later phase in which AMPK activity is reduced and rapid cell growth begins.

Although misregulation of calcium signaling can be important in tumorigenesis [131], direct regulation of tumor metabolism by calcium has not been studied in detail. Indeed, the role of AMPK in cancer metabolism is still controversial. While LKB1 has an established role as a tumor suppressor, LKB1 has a variety of substrates and how LKB1 tumor suppression relates to AMPK activation is unclear. AMPK activation has been proposed as being anti-tumorigenic, as it suppresses cell cycle progression and can oppose Akt activity by suppressing mTORC1 [132]. Recent data, however, indicates that transient AMPK activation in response to energy stress can promote tumor survival by maintaining NADPH homeostasis [133]. Understanding how AMPK activation supports activated T cells *in vivo* in times of metabolic stress may provide new clues as to the role of AMPK in tumor metabolism.

### Limitations of T cells as a model for tumor metabolism

Metabolic reprogramming in activated T cells is a useful model to study the metabolic changes that occur during tumorigenesis. Indeed, many of the pathways are similar and approaches to disrupt cancer metabolism can also be quite immunosuppressive. However, the two systems have some significant differences that may provide useful insight into novel anti-cancer therapies.

#### T Cell metabolic reprogramming is both transient and reversible

Following activation, T cells can differentiate into effector, regulatory and memory T cells that have differing metabolic profiles [2,117,134]. Activated T cells are, therefore, metabolically flexible and not fixed into a specific metabolic program. Unlike cancer cells with specific oncogenic mutations, T cell metabolism is dependent on signaling pathways triggered by the local environment. Indeed, even once T cell functional and metabolic fate has been defined there is a degree of reversibility and plasticity, for example, lipid-dependent regulatory T cells can be redirected to form highly glycolytic, IL-17-producing cells by altering the cytokine environment [41,135]. In contrast, tumor cells are largely fixed on one metabolic route that is dictated by irreversible mutations in upstream signaling pathways. Thus, cancer cells have less metabolic flexibility than T cells and the response of each cell type to inhibition of specific metabolic pathways may lead to distinctly different outcomes.

#### Activated T cells are not tumorigenic

Despite the metabolic and other similarities between stimulated T cells and a cancer cell undergoing aerobic glycolysis, activated T cells are not cancerous. Instead, following clearance of an infection the vast majority of activated T cells will die due to activation-induced cell death or from cytokine neglect. Both activated T cells and tumor cells are kept alive by a precarious balance of pro- and anti-apoptotic BH3 domain-containing proteins. In lymphocytes this balance is maintained by cytokine signaling through Akt and other pathways, and, in addition, by glycolytic flux [136-139]. Within tumors this balance is maintained both by glycolytic flux and oncogenic signaling. Understanding how activated T cells die following the loss of glycolytic flux and cytokine signals may provide insight into how anti-metabolites kill, or fail to kill, cancer cells.

#### Tumor cells are metabolically and genetically diverse

It is becoming evident that while the phenomena of aerobic glycolysis is common to many tumors, different cancer cells, potentially even within the same tumor, are metabolically diverse. Even within cell lines established from the same type of tumor there exists significant metabolic variation [140,141]. This heterogeneity can be representative of cancer stage or subtype, as in prostate and breast cancer. Given the strong dependence of T cells on glutamine, activated T cells represent a better model for glutamine-addicted tumors, for example those driven by oncogenic Myc [21,95], than more glucose dependent tumors, for example those driven by Met [141]. More importantly, activated T cells themselves become metabolically diverse as they differentiate into specific effector or regulatory subsets [41]. These T cell differentiation pathways are regulated by specific signaling events and it will be interesting to determine if distinct T cell subtypes may represent specific cancer types or stages. This is an important consideration as the sensitivity of tumor cells to metabolic inhibitors varies depending on the oncogenes involved [142].

## Conclusions

Cancer cells and activated T cells adopt comparable metabolic profiles to cope with similar environmental and proliferative stressors. Given that both T cell activation and tumorigenesis often resort to the same signaling pathways to induce this metabolic rewiring, T cell activation offers a useful model with which to study the mechanics of metabolic reprogramming. While cancer metabolism is inherently more diverse and susceptible to selective pressures, T cells have the significant advantage in a laboratory setting of being quiescent and non-cycling prior to activation, aiding in the delineation of cell signaling and cell cycle effects.

The aerobic glycolysis and glutamine dependency of cancer cells have been identified as potential novel targets for cancer therapy, and so developing an improved understanding of how these metabolic programs arise is of clinical importance. However, given the close similarity between activated T cell and tumor metabolic reprogramming, consideration must be given to the impact drugs targeting these pathways will have on T cells. T cell metabolism and T cell survival are intertwined, and the loss of anti-tumor T cells may negate many of the benefits of drugs targeting tumor metabolism. This is especially significant in the context of recent data indicating that metabolic suppression of activating T cells skews them toward an immunosuppressive phenotype, which may suppress anti-tumor immune responses [41].

## Abbreviations

acetyl-CoA: acetyl-CoenzymeA; AMPK: AMP activated protein kinase; APC: antigen-presenting cell; F26P2: fructose-2,6-bisphosphate; G6P: glucose-6-phosphate; HIF1α: hypoxia inducible factor 1α; HK: hexokinase; JAK: Janus kinase; LDH: lactate dehydrogenase; LDLR: low-density lipoprotein receptor; MAPK: mitogen-activated protein kinase; MCT: mono-carboxylic transporters; MHC: major histocompatibility complexes; mTORC1: mammalian target of rapamycin complex 1; NADPH: nicotinamide adenine dinucleotide phosphate; OXPHOS: oxidative phosphorylation; PFK1: 6-phosphofructo-1-kinase; PFKFB: 6-phosphofructo-2-kinase/fructose-2,6-bisphosphatase; PI(3,4,5)P3: phosphatidylinositol-3,4,5-trisphosphate; PI3K: phosphatidylinositol-3-kinase; PKM2: pyruvate kinase M2; PPP: pentose phosphate pathway; PTEN: phosphatase and tensin homologue deleted on chromosome 10; ROS: reactive oxygen species; SGLT: sodium/glucose linked transporter; SOCS: suppressor of cytokine signaling; STAT: signal transducer and activator of transcription; TCA: tri-carboxylic acid; TCR: T cell antigen receptor; TKTL1: transketolase 1; γc: common gamma chain.

## Competing interests

The authors declare that they have no competing interests.

## Authors’ contributions

ANM and JCR wrote the manuscript. Both authors read and approved the final manuscript.
